# Malignant inflammation in cutaneous T‐cell lymphoma—a hostile takeover

**DOI:** 10.1007/s00281-016-0594-9

**Published:** 2016-10-07

**Authors:** Thorbjørn Krejsgaard, Lise M. Lindahl, Nigel P. Mongan, Mariusz A. Wasik, Ivan V. Litvinov, Lars Iversen, Erik Langhoff, Anders Woetmann, Niels Odum

**Affiliations:** 1 Department of Immunology and Microbiology, University of Copenhagen, Blegdamsvej 3c, DK-2200 Copenhagen N, Denmark; 2 Department of Dermatology, Aarhus University Hospital, Aarhus, Denmark; 3 School of Veterinary Medicine and Science, University of Nottingham, Loughborough, UK; 4 Department of Pathology and Laboratory Medicine, University of Pennsylvania, Philadelphia, PA USA; 5 Division of Dermatology, University of Ottawa, Ottawa, ON Canada; 6 James J. Peters VA Medical Center, Veterans Affairs, Bronx, NY USA

**Keywords:** Cutaneous T-cell lymphoma, Malignant T cells, Inflammation, Pathogenesis, Cancer, Infection, Mycosis fungoides, Sézary syndrome

## Abstract

Cutaneous T-cell lymphomas (CTCL) are characterized by the presence of chronically inflamed skin lesions containing malignant T cells. Early disease presents as limited skin patches or plaques and exhibits an indolent behavior. For many patients, the disease never progresses beyond this stage, but in approximately one third of patients, the disease becomes progressive, and the skin lesions start to expand and evolve. Eventually, overt tumors develop and the malignant T cells may disseminate to the blood, lymph nodes, bone marrow, and visceral organs, often with a fatal outcome. The transition from early indolent to progressive and advanced disease is accompanied by a significant shift in the nature of the tumor-associated inflammation. This shift does not appear to be an epiphenomenon but rather a critical step in disease progression. Emerging evidence supports that the malignant T cells take control of the inflammatory environment, suppressing cellular immunity and anti-tumor responses while promoting a chronic inflammatory milieu that fuels their own expansion. Here, we review the inflammatory changes associated with disease progression in CTCL and point to their wider relevance in other cancer contexts. We further define the term “malignant inflammation” as a pro-tumorigenic inflammatory environment orchestrated by the tumor cells and discuss some of the mechanisms driving the development of malignant inflammation in CTCL.

## Introduction

**Fig. 1:**
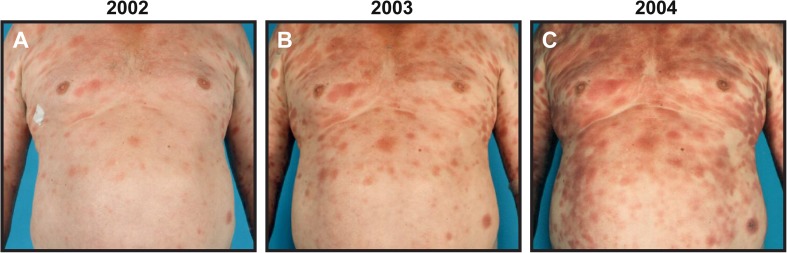
Progressive MF. **a**–**c** Example of a patient with progressive MF that during a 3-year period (2002–2004) presents with increasing skin involvement and inflammation. The patient progressed from **a** clinical stage IA disease with limited patches and plaques to **b**, **c** clinical stage IB disease with patches and plaques involving more than 10 % of the skin surface area

Cutaneous T-cell lymphomas (CTCL) are characterized by the presence of malignant T cells in chronically inflamed skin lesions. Mycosis fungoides (MF) is the predominant clinical variant comprising 54–72 % of all cases [1–3]. Classically, MF presents as erythematous skin patches bearing strong resemblance to benign inflammatory dermatoses. Early disease, which is characterized by limited patches or plaques (Fig. 1a), typically exhibits an indolent behavior and a very favorable prognosis with normal life expectancy [1–3]. For many patients, the disease never progresses beyond this stage, but for about one third of patients the skin lesions start to spread (Fig. 1b, c) and, eventually, overt tumors and generalized erythroderma may develop [2]. In advanced disease, the malignant T cells can further disseminate to the lymphatic system, blood, bone marrow, and internal organs. There is a stage-dependent decrease in survival, and patients with late stages of CTCL have a median life expectancy of less than 4 years [1–5]. The risk of disease progression rises significantly as the disease advances highlighting that it becomes increasingly aggressive [4]. Sézary syndrome (SS) is a particular aggressive variant of CTCL that can arise de novo or, rarely, ensue after years of chronic MF [1, 4–6]. The characteristics of SS include generalized erythroderma, lymphadenopathy, and peripheral blood involvement, classifying SS as an advanced clinical stage of CTCL [1, 2, 6]. Because MF and SS are the best studied forms of CTCL and collectively comprise the majority of cases, the term CTCL will in the following generally refer to these two clinical variants [1, 2].

The malignant T cells typically display the phenotype of CD4^+^ memory T lymphocytes and express skin-homing receptors such as cutaneous lymphocyte antigen (CLA), CC chemokine receptor 4 (CCR4), and CCR10 [1, 7–11]. In low-grade MF, the malignant T cells also express CXC chemokine receptor 3 (CXCR3), which promotes their recruitment to the dermis and epidermis where the corresponding ligands (CXCL9, CXCL10, and CXCL11) are synthesized [8, 12–14]. Based on the expression of CCR7, L-selectin, and CD27, it has further been reported that the phenotype of the malignant T cells resembles that of skin resident effector memory T cells in MF and that of central memory T cells in SS [9]. However, many characteristics of the malignant T cells change during disease progression, and there seems to be a relatively high degree of heterogeneity and plasticity within the malignant population [15].

The etiology of MF and SS remains an enigma. Demographic patterns in the incidence of CTCL have been reported, but no environmental triggers have yet been identified [16, 17]. Likewise, no convincing evidence showing an etiologic role of infectious agents in CTCL has been provided, albeit infections may aggravate the disease [18]. A recent series of studies profiled the genomic landscape of CTCL [19–25]. Collectively, the results demonstrated that the genomic landscape is very heterogeneous, and that the disease is not driven by a few well-defined somatic mutations, copy number variations, or fusion proteins. Somatic genetic alterations were, however, frequently found in genes involved in specific cellular processes and signaling pathways, including epigenetic regulation, DNA damage response, cell cycle control, programmed cell death, T cell receptor (TCR) signaling, as well as the nuclear factor-kappa B (NF-κB) and Janus kinase (Jak)/signal transducer and activator of transcription (Stat) pathways [19–25]. Accordingly, numerous studies have previously raised these cellular processes and pathways as key players in the pathogenesis of CTCL. Three studies found a high prevalence of ultraviolet (UV) light signature mutations in patients with MF and leukemic CTCL [19, 22, 25]. No correlation between the presence of the UV light signature and previous therapeutic UV light exposure was observed in leukemic patients, arguing that accumulated lifetime exposure to natural UV light induced the UV mutational signature, thereby challenging the view that the disease originates from central memory T cells rather than skin resident memory T cells [19, 22].

**Fig. 2:**
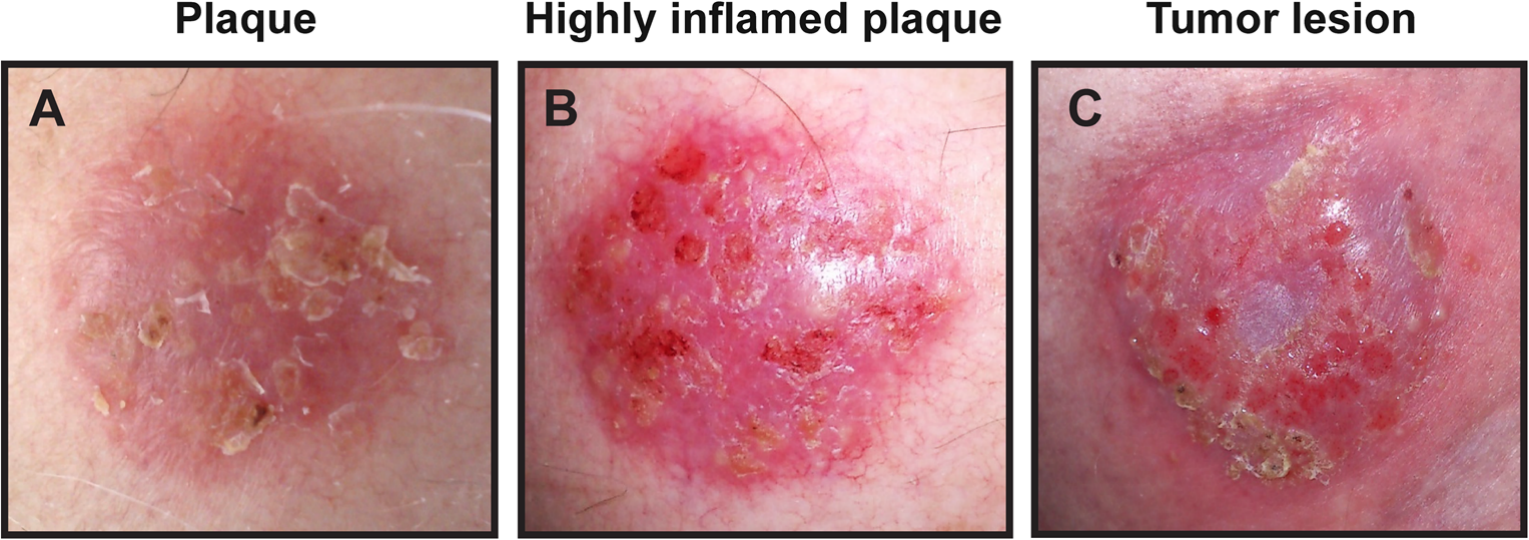
MF lesions display different levels of inflammation. **a**–**c** Illustrative skin lesions from a patient with MF displaying varying degrees of inflammation

Despite stage-appropriate treatment, a significant subset of CTCL patients, eventually, develop progressive disease [2, 26]. Given the profoundly reduced survival and increased clinical aggressiveness of advanced CTCL, it is of outmost importance to map the mechanisms that underlie disease progression [4, 5]. A better understanding of these mechanisms may lead to the development of more efficient strategies to diagnose, prevent and treat progressive disease. CTCL lesions can display varying degrees of inflammation (Fig. 2), and a large number of studies have highlighted changes in the tumor-associated inflammatory environment as a critical checkpoint in the transition from early indolent to progressive and advanced disease. Here, we review the inflammatory changes associated with disease progression in CTCL and outline some of the mechanisms that appear to drive the development of malignant inflammation—a pro-tumorigenic inflammatory environment orchestrated by the tumor cells.

## The inflammatory environment becomes Th2-biased during the clinical course of CTCL

In early disease stages, the skin lesions contain of a small population of malignant T cells immersed within a dense infiltrate of reactive immune cells. A significant proportion of the immune cells are activated CD8^+^ T cells and T helper 1 (Th1) cells expressing cytotoxic molecules, implying that early inflammation encompasses a cell-mediated anti-tumor response that actively suppresses the expansion of the malignant cells [27–33]. Indeed, reactive CD4^+^ T cells, CD8^+^ T cells and natural killer (NK) cells isolated from CTCL patients are able to kill autologous malignant T cells in vitro, and high numbers of CD8^+^ lymphocytes in skin lesions and/or blood are linked to a favorable prognosis [30, 32, 34–37].

Disease progression is, however, associated with increasing expression of Th2 markers (e.g., GATA-3) and cytokines (e.g. interleukin (IL)-4, IL-5, and IL-13) concomitant with declining expression of Th1 factors such as T cell-specific T-box transcription factor (T-bet), interferon gamma (IFN-γ), Stat4, and IL-12 [33, 38–43]. Accordingly, late-stage CTCL is characterized by a Th2-dominated tumor microenvironment and a paucity of benign Th1 cells and CD8^+^ T cells [32–34, 37–39, 41]. The growing Th2-bias is believed to be a key process suppressing cellular immunity and anti-tumor responses in CTCL, and the impairment of the immune defense can especially be seen in patients with advanced disease who often suffer from severe and sometimes fatal bacterial infections [27, 44].

## Changes in the chemokine profile facilitate the development of a Th2-biased tumor microenvironment and direct the trafficking of the malignant T cells

Parallel to the shift in the balance of Th1 and Th2 cytokines, there is a decrease in chemokines that preferentially attract Th1-associated inflammatory cells, such as CXCL9 and CXCL10, and an increase in chemokines that primarily attract Th2-associated inflammatory cells [14]. For example, Miyagaki et al. demonstrated that fibroblasts in early CTCL lesions express high levels of the chemokines CXCL9 and CXCL10, which preferentially attract Th1 cells, CD8^+^ T cells, and NK cells [45, 46]. Secretion of these chemokines was triggered in explanted CTCL skin fibroblasts upon co-stimulation with IFN-γ and LIGHT (TNFSF14). Whereas the expression of LIGHT was increased in advanced when compared with early disease, the expression of its receptor, herpesvirus entry mediator (HVEM), was decreased. The authors found a direct correlation between the expression levels of CXCL9, CXCL10, and HVEM, indicating that downregulation of HVEM and IFN-γ, at least partly, is responsible for the decreased expression of CXCL9 and CXCL10 during disease progression [45]. Conversely, the concentrations of Th2-associated chemokines such as CCL17, CCL18, CCL22, and CCL26 have been shown to increase from early to advanced disease [47–50]. These chemokines are primarily secreted by diverse non-malignant cell types within the skin lesions, and by favoring recruitment of Th2 cells relative to that of Th1 cells, most likely play a central role in CTCL progression through promoting a Th2-dominated inflammatory microenvironment [7, 46, 47, 50–53].

In early disease, the malignant T cells express CXCR3, the receptor for CXCL9 and CXCL10, and the CXCR3-CXCL9/CXCL10 axis has been proposed to be important for the dermal and epidermal accumulation of the malignant T cells at this stage. However, similar to CXCL9 and CXCL10, the expression of CXCR3 also decreases during disease progression, which is accompanied by a gradual loss of epidermotropism [12, 13, 45]. In contrast, CCR4, the receptor for CCL17 and CCL22, is continuously expressed by the malignant T cells and, in line with the upregulation of CCL17 and CCL22, the number of CCR4-positive T cells increases in later disease stages [10, 47, 49]. A recent phase I/II clinical study of the humanized anti-CCR4 antibody, mogamulizumab, showed promising results in heavily pretreated patients, supporting an important role of the CCR4-CCL17/CCL22 axis in the pathogenesis of CTCL [54]. In advanced disease stages, the expression of the chemokine receptor CCR6 and its ligand CCL20 is increased in the lesional skin [55, 56]. Of interest, Ikeda and co-workers reported that disruption of CCL20-CCR6 interactions inhibited disease spread in NOG mice inoculated with CCR6^+^CCL20^+^ malignant CTCL cells, arguing that upregulation of the CCR6-CCL20 axis might promote malignant dissemination in some cases [57]. Collectively, these findings emphasize that the expression of chemokines and chemokine receptors is dynamic during the progression of CTCL, and that distinct chemokine-chemokine receptor interactions shape the inflammatory microenvironment and the trafficking of malignant T cells at different disease stages.

## The expression of pro-tumorigenic cytokines increases during disease progression

Besides the rise in Th2-associated cytokines and chemokines, the expression of a range of cytokines, including IL-10, IL-15, IL-16, IL-17A, IL-17F, IL-22, and IL-32 also increases during the clinical course of the disease [55, 58–65]. As briefly described below, all of these cytokines have been reported to play pathogenic roles in CTCL. For instance, IL-10 was shown to suppress Th1 cytokine production by CTCL cells, and malignant CTCL cells impede dendritic cell maturation, as well as activation of benign T cells, in an IL-10-dependent manner, indicating that IL-10 contributes to the suppression of cellular immunity and anti-tumor responses [66–68]. IL-16 is a growth factor and a chemo-attractant for CD4^+^ T cells. Accordingly, evidence supports that elevated levels of IL-16 contribute to the cutaneous accumulation of the malignant T cells by augmenting their intradermal proliferation and potentiating their recruitment into the skin [49, 69]. The expression of IL-17A and IL-17F is heterogeneous and confined to a subset of patients which is likely the cause of discrepant reports regarding their expression in CTCL [55, 62, 70–73]. High expression of IL-17F has been associated with significantly increased risk of disease progression independently of disease stage, sex, and age at the time of diagnosis and IL-17A with neutrophil infiltration in CTCL lesions [62, 70]. In agreement, marked infiltration of neutrophils is only observed in a subset of CTCL patients and their accumulation has been associated with an aggressive disease course [70, 74]. IL-22 has been reported to correlate with the expression of CCL20 in CTCL lesions, and siRNA-mediated knockdown of IL-22 receptor alpha (IL-22RA) in malignant CTCL cells inhibited the expression of CCL20, providing a possible link between IL-22, the CCR6-CLL20 axis, and malignant dissemination [55, 56]. Finally, IL-32 has been shown to promote the proliferation and survival of malignant CTCL cells via NF-κB- and mitogen-activated protein kinase (MAPK)-dependent mechanisms [64, 65].

Taken together, a plethora of distinct cytokines and chemokines are upregulated during the progression of CTCL and seem to facilitate expansion of the malignant T cells. Disease progression, thus, appears to be associated with a shift in the nature and cellular composition of the tumor-associated inflammatory environment from partly anti-tumorigenic to pro-tumorigenic. This raises the key question—what is driving these inflammatory changes? Are the changes an epiphenomenon of a frustrated “host” anti-tumor immune response, or are they an important ingredient of a cancer-cell-driven escape from the “host” immunity and a result of a “parasitic” exploitation of the microenvironment orchestrated by the malignant T cells?

## The malignant T cells promote the development of a lesional Th2-bias

Providing a pivotal clue to this question, several reports have shown that the malignant T cells in advanced stages express high levels of the Th2-cytokines IL-4, IL-5, and IL-13 and low levels of IFN-γ [41, 75, 76]. At the molecular level, the skewed Th2 cytokine profile, at least partly, seems to be mediated by a progressive dysregulation of the Jak/Stat pathway and increased expression of the Th2-lineage-specific transcription factor GATA-3 [33, 43]. Under normal conditions, Stat proteins are only transiently activated but genetic alterations, epigenetic changes, persistent cytokine signaling, and infections appear to drive constitutive activation of Stat3, Stat5, and Stat6 signaling in the malignant T cells during the clinical course of CTCL [19, 21, 24, 41, 43, 77–83]. Aberrant activation of these transcription factors not only fosters the growth and survival of the malignant T cells but also stimulates the secretion of Th2 cytokines [43, 75, 80]. Expression of Stat4, which promotes Th1 differentiation and IFN-γ expression, is on the contrary very frequently lost in advanced disease [42, 43, 84–86]. Evidence suggests that aberrant activation of Stat5 in the malignant T cells induces expression of miR-155 which subsequently inhibits the expression of Stat4 [84, 87, 88]. The mechanisms underlying the increased expression of GATA-3 in the malignant T cells have not yet been elucidated. However, it is noteworthy that several large-scale genomic studies recently found that the *ZEB1* gene, which is a potent transcriptional repressor of GATA-3, is somatically targeted by deleterious mutations or deleted in 45–65 % of patients with advanced CTCL [19, 21, 22, 25].

It is well established that Th1 cytokines enforce Th1- and repress Th2-mediated inflammation and vice versa, suggesting that the phenotypic shift of the malignant T cells towards a Th2 profile might instigate the development of the generalized Th2-bias in CTCL lesions. Indeed, a recent study by Guenova et al. demonstrated that benign T cells isolated from patients with leukemic CTCL displayed reduced Th2 and enhanced Th1 responses when cultured separately from the malignant T cells [76]. Likewise, T cells from healthy donors demonstrated significantly reduced ability to secrete IFN-γ when co-cultured with leukemic CTCL cells. The malignant T cell-induced suppression of IFN-γ production by the healthy T cells was completely blocked by neutralizing antibodies against IL-4 and IL-13. Notably, separate culture had no effect on the production of Th1 and Th2 cytokines by isolated malignant T cells. The authors further addressed how treatment with a variety of modalities, including UVB phototherapy, extracorporal photopheresis, low-dose alemtuzumab, and systemic chemotherapy with gemcitabine influenced the frequency of benign T cells expressing IFN-γ and IL-4 in leukemic CTCL patients [76]. In line with the in vitro results, they found that in-spite of distinct mechanisms of action, all treatment modalities that successfully reduced the malignant T cell burden strongly increased the frequency of IFN-γ-expressing, and decreased the frequency of IL-4-expressing, benign T lymphocytes [76]. Collectively, these findings imply that progressive dysregulation of the Jak/Stat pathway and upregulation of GATA-3 in the malignant T cells lead to their increased synthesis of IL-4 and IL-13 which suppresses benign Th1 responses and promotes a generalized Th2-bias.

Malignant T cells may also contribute indirectly to the shifting Th1/Th2 balance by regulating the expression of chemokines within the lesional skin. Whereas IFN-γ preferentially induces expression of CXCL9 and CXCL10, IL-4 and IL-13 primarily induce expression of CCL17, CCL18, CCL22, and CCL26 [46, 89–94]. It is therefore plausible that the increased expression of Th2 cytokines and decreased expression of Th1 cytokines by the malignant T cells create a positive feedback loop by promoting the secretion of Th2 chemokines from benign cells in the tumor microenvironment (e.g., tumor-associated macrophages, fibroblasts, and keratinocytes). This, in turn, favors the recruitment of Th2 cells, ultimately, leading to enhanced expression of Th2 and decreased expression of Th1 cytokines. Accordingly, significant correlations between the expression of IL-4 and CCL18, as well as IL-4 and CCL26, in CTCL skin lesions were previously reported [48, 50].

## The malignant T cells suppress anti-tumor immunity via cell contact-dependent and cell contact-independent mechanisms

The malignant T cells may, however, not only suppress anti-tumor immunity by modulating the nature of the inflammatory microenvironment but can also directly kill or suppress the activation and proliferation of benign immune cells. For example, aberrant activation of Stat5 has been shown to induce expression of the B7 family member, CD80 (B7-1), on the surface of malignant CTCL cells [95]. CD80 is an immunoregulatory molecule that can deliver growth-inhibitory signals to activated T cells via the receptor CD152 (CTLA-4) [96]. Whereas depletion of CD80 in the malignant T cells did not influence their proliferation or viability, the malignant T cells inhibited the proliferation of benign T cells in a CD152- and CD80-dependent manner [95]. The Jak/Stat pathway was, likewise, proposed to induce malignant T cell expression of another inhibitory B7 family member, namely PD-L1 (B7-H1), which has been implicated in benign T cell suppression and tumor immune evasion in CTCL [97–100]. Interestingly, it was demonstrated that the malignant T cells can be targeted by PD-L1-specific cytotoxic T cells, indicating that the immune system is able to react to immune escape mechanisms of the tumor cells [101]. The malignant T cells also frequently express high levels of Fas ligand (FasL) which can induce apoptosis by engaging the death receptor, Fas, on target cells [102]. Malignant CTCL cells have been shown to induce FasL-mediated T cell apoptosis in vitro, and the numbers of CD8^+^ T cells are inversely distributed with FasL-positive tumor cells in situ, supporting that the malignant T cells utilize FasL to kill tumor-reactive T cells [102, 103]. Due to loss of Fas expression, somatic inactivating mutations and deletions of the *FAS* and *ARID1a* genes or increased expression of anti-apoptotic proteins such as c-FLIP, the malignant T cells typically develop resistance to FasL-mediated apoptosis which contrarily protects them from activation-induced apoptosis and T cell-mediated cytotoxicity [19, 21, 22, 104–107].

In addition to these cell contact-dependent mechanisms, the malignant T cells may also directly suppress the immune system via secretion of soluble factors. Aberrant activation of the Jak/Stat pathway has, for example, been shown to induce expression of the immunoregulatory cytokines IL-10 and transforming growth factor beta (TGF-β) in malignant CTCL cells [68, 108]. As noted above, malignant CTCL cells impair DC maturation and activation of benign T cells in an IL-10-dependent manner. Similarly, TGF-β was reported to enhance the malignant T cells’ ability to inhibit activation of syngenic and allogenic normal T cells [66–68, 109]. Given the potent immunosuppressive capacities of IL-10 and TGF-β, it could be expected that they would also inhibit the proliferation and cytokine production of the malignant T cells in an autocrine fashion. However, as observed with the expression of Fas, malignant T cells in advanced CTCL frequently lack expression of functional receptors and/or downstream signaling mediators for IL-10 and TGF-β, essentially rendering them resistant to the suppressive activities of these cytokines [82, 86, 110, 111]. Downregulation of Zeb1 has, notably, been shown to confer resistance to TGF-β-mediated growth inhibition in malignant T cells from adult T-cell leukemia/lymphoma [112]. These findings exemplify that malignant T cells are able to simultaneously exploit and escape suppressive mechanisms normally deployed by the immune system. In a similar fashion, aberrant activation of Stat3 induces constitutive expression of suppressor of cytokine signaling-3 (SOCS3), which attenuates the effects of several cytokines, including IFN-γ and IFN-α and, consequently, protects the malignant T cells from cytokine-mediated anti-tumor responses [113]. Altogether, these data clearly indicates that the malignant T cells become resistant to immunosuppressive factors (e.g., dysfunction of Fas-, IL-10-, and TGF-β-signaling and upregulation of SOCS3) while acquiring the capacity to inhibit anti-tumor immunity by means of cell contact-dependent (e.g., CD80, PD-L1, and FasL) and independent (e.g., Th2 cytokines, IL-10, TGF-β) mechanisms.

## The malignant T cells produce autocrine growth factors that activate pro-oncogenic pathways

The development of a Th2-dominated tumor microenvironment may not merely impede cellular immunity and anti-tumor responses but also directly fuel the growth of the malignant T cells. Intriguingly, a recent study by Geskin et al. provided evidence that the malignant T cells express functional receptors for IL-4 and IL-13, and that these cytokines promote the activation of Stat6 and the proliferation of the tumor cells [41]. Accordingly, neutralizing antibodies against IL-4 and IL-13, as well as a Stat6 inhibitor, significantly retarded the proliferation of CD4^+^ T cells isolated from leukemic CTCL patients [41]. These findings imply that IL-4 and IL-13 produced by malignant T cells and benign Th2 cells stimulate the proliferation of the malignant T cells via activation of Stat6. Other cytokines that are upregulated in advanced disease, including IL-15, IL-16, and IL-32, can also augment the growth of the malignant T cells in an autocrine manner [49, 59, 61, 64, 65, 114]. Of particular interest, IL-15 activates Stat5 and Stat3 in malignant CTCL cells and has been proposed to contribute to the aberrant activation of these transcription factors [43, 108, 115]. A possible explanation for the increased expression of IL-15 was provided by a recent study which documented that the promoter region where Zeb1 can bind and repress transcription of IL-15 is hypermethylated in malignant T cells from leukemic CTCL patients, resulting in impaired binding of Zeb1 and unchecked IL-15 expression [60]. This finding implicates Zeb1 as a putative tumor suppressor of several pathogenic processes in CTCL and suggests that in patients in whom the *ZEB1* gene is not mutated, tumor suppressive functions of the Zeb1 protein may instead be impeded by other mechanisms such as epigenetic modification of its target DNA. Interestingly, malignant T cells are not the only source of IL-15 in the lesional skin, as stromal cells also have the capacity to express IL-15. In particular, epidermal keratinocytes that become activated by malignant T cells in vitro and in vivo, have been shown to express IL-15 in situ in CTCL lesions, indicating that malignant T cells may also propagate their own proliferation through stimulation of keratinocytes to produce tumor growth factors such as IL-15 [114, 116]. The fact that transgenic mice overexpressing IL-15 develop a neoplasm that mimics human CTCL reinforces the idea that IL-15 plays an important role in the disease pathogenesis [60].

Constitutive activation of the transcription factor NF-κB is, like dysregulation of the Jak/Stat pathway, a characteristic feature of malignant CTCL cells and numerous reports have demonstrated that NF-κB promotes their proliferation and survival [117, 118]. It was recently reported that IL-32 stimulates the growth of malignant CTCL cells in a NF-κB-dependent manner. This finding provides circumstantial evidence that upregulation of IL-32 during disease progression may contribute to the constitutive activation of NF-κB [65]. Altoghether, these data illustrate that in advanced disease the malignant T cells produce cytokines (e.g., IL-4, IL-13, IL-15 and IL-32), which can induce chronic activation of key pro-oncogenic signaling pathways in an autocrine manner. The arsenal of autocrine growth factors that the malignant T cells are able to produce is not limited solely to cytokines. For example, it has been shown that the malignant T cells begin to express cyclooxygenase-2 (COX-2) in plaque-stage disease, and that the expression of this protein correlates with in vitro secretion of its downstream product, prostaglandin E2 (PGE2) [119]. Selective knockdown of COX-2 or PGE2 receptor antagonists inhibited the spontaneous proliferation of the malignant T cells in vitro [119]. Furthermore, the COX-2 inhibitor, celecoxib, reduced tumor growth in a xenograft model of advanced MF, implicating PGE2 as an autocrine growth factor in CTCL [120]. PGE2 is known to hamper cell-mediated immunity by various mechanisms. These include inhibition of Th1 cytokine production, repression of CXCL9 and CXCL10 expression as well as suppression of NK cell- and CD8^+^ T cell-mediated cytotoxicity [121]. Hence, analogous to IL-4 and IL-13, PGE2 may have a dual function in malignant inflammation by hampering cellular immunity while directly stimulating the growth of the malignant T cells. In summary, the malignant T cells appear to gain the capacity to produce a spectrum of cytokines and other inflammatory factors that can promote activation of pro-oncogenic pathways and stimulate their own expansion.

## The malignant T cells foster pro-tumorigenic inflammation through interactions with stromal and innate immune cells

In line with the growing Th2-bias and malignant T cell expression of factors such as PGE2, IL-10, and high-mobility group BOX-1 protein (HMGB1), the infiltration of mast cells, eosinophils, and tumor-associated macrophages (TAMs) with an M2 phenotype, typically increases during CTCL progression [89, 122–127]. A high degree of infiltration by each of these immune subsets has been linked to poor prognosis, suggesting that the malignant T cells may foster a pro-tumorigenic environment by promoting accumulation of granulocytes and TAMs [122, 123, 127]. Accordingly, Rabenhorst et al. demonstrated that mast cells in CTCL lesions generally exhibit a degranulated phenotype, and that their numbers correlate with the microvessel density [122]. Supernatant from activated mast cells was further able to promote the proliferation of malignant CTCL cells in vitro, and depletion of mast cells in a murine T cell lymphoma model inhibited tumor growth, strongly supporting that mast cells play a pro-tumorigenic role in CTCL [122]. In a similar manner, eosinophils often display an activated phenotype and express IL-5 in CTCL skin lesions, indicating that they release inflammatory factors into the tumor microenvironment that may aggravate the disease [123]. Indeed, accumulation of eosinophils in blood and lesional skin of CTCL patients has not only been associated with poor prognosis but also with increased pruritus [6]. Activated eosinophils can, in addition to IL-5, release high levels of IL-4 and IL-13 as well as angiogenic factors. This raises the possibility that malignant T cells, through the attraction and activation of eosinophils, may fuel their own growth indirectly by boosting neovascularization and a deregulated Th2 response [128]. Marked infiltration of neutrophils is only occasionally seen in CTCL, but detection of neutrophilic dermatoses has been associated with an aggressive disease course [70, 74]. As already mentioned, L-17A and IL-17F are expressed in the lesional skin in a subset of CTCL patients and known to stimulate recruitment of neutrophils [62, 70, 71]. High expression of IL-17A has, accordingly, been associated with neutrophilic infiltration into CTCL lesions; whether it is also the case for IL-17F remains to be investigated [70]. Malignant T cells derived from some patients have the capacity to express IL-17A and/or IL-17F via a Jak3/Stat3/Stat5-dependent mechanism but do not express the Th17-linage transcription factor, RORc, or display a characteristic Th17-phenotype [62, 71, 76, 129]. Therefore, expression of IL-17A and IL-17F by the malignant T cells is probably not reflective of Th17 origin, or acquisition of a full-fledged Th17 phenotype, but rather a consequence of dysregulated signaling. Several cell types, including Th17 cells, mast cells and neutrophils are also known to produce IL-17 cytokines and it is likely that they also provide a significant source of IL-17A and IL-17F in some lesions. Accordingly, skin-infiltrating neutrophils and mast cells have been reported to stain positive for IL-17 in CTCL patients with neutrophilic dermatoses [72].

The malignant T cells can also exploit monocytes and dendritic cells to stimulate their expansion. For example, malignant CTCL cells engage in cell contact-dependent crosstalk with immature dendritic cells ex vivo that results in prolonged survival and proliferation of both the malignant T cells and the immature dendritic cells. The malignant T cells have, interestingly, been shown to arrest dendritic cells in an immature state via secretion of regulatory cytokines such as IL-10, indicating that they block dendritic cell maturation to perpetuate their own growth and maintain a tolerogenic environment [67, 130].

M2-like TAMs are generally believed to contribute to tumorigenesis by promoting angiogenesis, matrix remodeling and inhibition of adaptive immunity [131]. In agreement with the notion that the malignant T cells drive the accumulation of M2-like TAMs during the clinical course of CTCL, M2-like macrophages were found to be abundant within the tumor microenvironment, but not in the spleen, in a CTCL mouse model [126]. Furthermore, effective treatment of CTCL patients resulting in decreased numbers of tumor cells was shown to be accompanied by a strong decrease of dermal TAMs in the lesional skin [127]. TAMs express CCL18 and MMP9 in CTCL lesions, and clodronate-mediated depletion of M2-like macrophages in a mouse model of CTCL inhibited angiogenesis, lymphangiogenesis, and tumor development. Collectively, these results implicate M2-like TAMs in vascularization, chemotaxis, tissue remodeling, and tumor growth in CTCL [51, 126, 132].

The malignant T cells also display aberrant release of cytokines and other factors, such as vascular endothelial growth factor (VEGF), prostaglandins, and lymphotoxins, which promote activation of endothelial cells and fibroblasts, thereby stimulating angiogenesis via both direct and indirect mechanisms [62, 70, 71, 81, 119, 133, 134]. For example, the malignant T cells can trough release of VEGF-A and lymphotoxin α induce enhanced endothelial sprouting in vitro and further trigger fibroblast expression of VEGF-C and angiogenesis in vivo*,* supporting the concept that the malignant T cells orchestrate profound changes in the tumor microenvironment [133, 135]. Indeed, the malignant T cells strongly impacted skin structure, as well as keratinocyte activation and proliferation, in an organotypic skin model and a xenograft CTCL mouse model [116]. Hence, malignant T cells and their supernatants triggered Jak and MAPK activation in keratinocytes, downregulation of differentiation markers such as keratin 10 and involucrin, epidermal hyperproliferation, disorganized keratinocyte stratification, and decreased barrier formation, thus mimicking many features seen in CTCL lesions [116]. In conclusion, accumulating evidence indicates that the malignant T cells, via interactions with stromal and benign immune cells, drive the disease stage-related inflammatory changes observed in CTCL.

## Drugs that stimulate cellular immunity induce disease regression

As highlighted by the multiple pieces of evidence outlined above, the shift from a Th1- towards a Th2-dominated tumor-associated inflammatory environment appears to play a central role in malignant T cell proliferation and tumor promotion and, at the same time, suppression of cell-mediated immunity and anti-tumor responses in CTCL. Consistent with this conclusion, administration of the Th1 cytokines IL-12 and IFN-γ can induce lesional regression associated with increased numbers of cytotoxic CD8^+^ T cells in the resolving skin, and treatment with Toll-like receptor (TLR) agonists that stimulate cellular immunity have shown clinical efficacy in CTCL patients [136–144]. For example, a recent clinical phase 1 trial reported that 75 % of the enrolled CTCL patients had significant improvement in skin lesions treated topically with resiquimod gel (TLR-7 and TLR-8 agonist) and 30 % had clearing of all treated lesions. Some patients even had a systemic response with improvement of untreated lesions. The responding patients displayed profoundly decreased numbers of malignant T cells, and high responses were associated with recruitment and expansion of benign T cell clones in treated skin, increased skin T cell effector functions and a trend towards increased NK cell function [144]. The fact that immunomodulatory drugs, which stimulate the cellular immune system, can induce lesional regression and promote eradication of the malignant T cells strongly supports that suppression of the patients’ cellular immunity represents a critical checkpoint for the expansion of the malignant T cells.

## Enterotoxin-producing *S. aureus* may promote malignant inflammation in CTCL

Aberrant activation of the Jak/Stat pathway seems to be a central event in the development of malignant inflammation in CTCL. A significant proportion of patients with advanced disease, accordingly, display genetic alterations that directly or indirectly promote constitutive activation of the Jak/Stat pathway; however, it is unclear what drives constitutive activation of Jak/Stat signaling in other patients [19, 21, 24, 25]. Due to a compromised cutaneous barrier and evolving immune dysfunction, the lesional skin of CTCL patients is often colonized with enterotoxin-producing *Staphylococcus aureus* which represent a major clinical problem [18, 44, 145–147]. Recent reports have demonstrated that staphylococcal enterotoxins (SE) instigate crosstalk between the malignant and benign T cells which leads to increased proliferation, activation of Stat3 and expression of Stat3-regulated cytokines in the malignant T cells [82, 83, 148]. Consequently, colonization of CTCL lesions with SE-producing *S. aureus* may trigger mechanisms that aggravate the disease and promote malignant inflammation. In support of this theory, a series of smaller studies and case reports have shown that antibiotic treatment, leading to successful elimination of *S. aureus*, is associated with significant clinical improvement in most colonized patients. Strikingly, in some patients, antibiotic treatment even resulted in complete clinical response with no residual skin involvement [18, 145, 146, 149, 150]. Evidence suggests that the malignant T cells are responsible for the impairment of the skin barrier in CTCL [116]. This indicates that the malignant T cells both induce increased susceptibility towards *S. aureus* and subsequently exploit the infection to initiate a vicious crosstalk with the benign T cells which results in activation of pro-oncogenic pathways. These pathways, in turn, promote the proliferation of the malignant T cells and their expression of pro-tumorigenic factors. Although the proposed mechanism is specific for SE-producing bacteria, it is likely that other infectious agents and extrinsic factors that impact the inflammatory environment may promote malignant inflammation and disease progression. On the other hand, it can be speculated that certain bacteria might be beneficial through their ability to regulate inflammation or out-compete disease-promoting bacteria and, thus, such bacteria may be of therapeutic use in CTCL if identified.

## Malignant inflammation—a hostile takeover

**Fig. 3:**
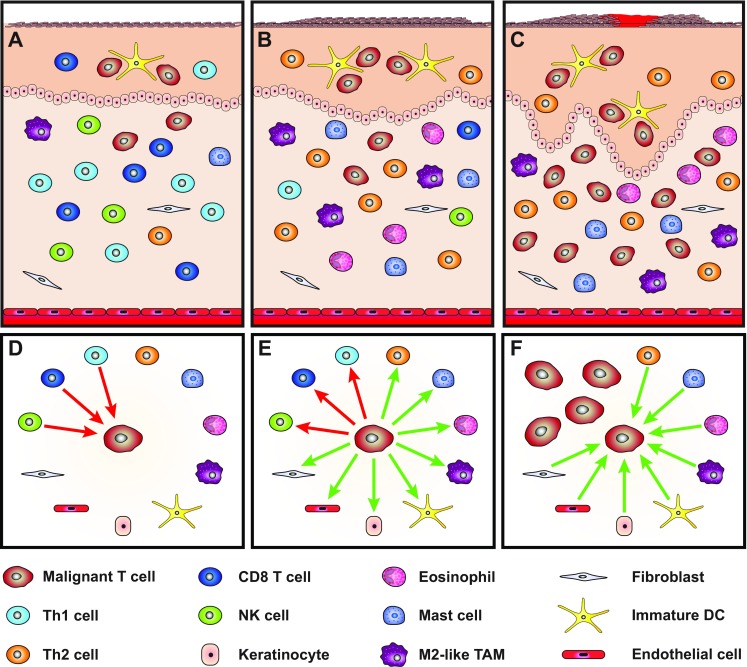
A model of malignant inflammation. **a**–**c** Schematic illustration of progression from **a** early indolent CTCL with few malignant T cells **b**, **c** to more advanced disease increasingly dominated by Th2-biased inflammation and malignant T cells. **d** In early indolent disease, the expansion of the malignant T cells is kept in check by a cellular anti-tumor immune response involving CD8^+^ T cells, Th1 cells, and NK cells. **e** In time, malignant T cell endogenous (e.g., genetic and epigenetic alterations) and exogenous (e.g., irradiation, toxins, microbes, chemicals, drugs) events can enable the malignant T cells to take control of the tumor microenvironment, thereby inhibiting the cellular anti-tumor immune response while stimulating the accumulation and/or activation of certain types of benign immune cells and stromal cells (**f**) that produce pro-tumorigenic factors which directly or indirectly foster the survival and expansion of the malignant T cells

The inflammatory reaction in early indolent disease appears to be a “host” response directed against the malignant T cells and potentially other factors in the tumor microenvironment. Early inflammation, accordingly, involves CD8^+^ T cells, Th1 cells and possibly NK cells in conjunction with cytokines that inhibit the expansion of the malignant T cells. However, the nature and cellular composition of the tumor-associated inflammatory environment seem to shift from partly anti-tumorigenic to pro-tumorigenic during disease progression. As reviewed above, considerable evidence suggests that the malignant T cells orchestrate the shift in the inflammatory microenvironment, enforcing profound changes in the cytokine and chemokine milieu as well as in the composition, activity and function of most cellular components within the cutaneous lesions. These changes, in turn, promote tumorigenic processes, including angiogenesis, lymph-angiogenesis, tissue remodeling, and tumor growth. The malignant T cells, thus, appear to perform a “hostile” takeover of the lesional microenvironment, thereby gaining control of the tumor-associated inflammation to create a setting that facilitates their own expansion (Fig. 3). The hostile takeover is exemplified by the tumor cell-mediated induction of (i) malignant growth factors such as IL-4, IL-13, and IL-15 from non-malignant cells, (ii) M2-like TAMs, mast cells, and immature dendritic cells that provide growth and survival signals to the malignant T cells, and (iii) angiogenesis via stimulation of fibroblasts, innate immune cells and endothelial cells. In other words, malignant T cells engage in a “parasitic” exploitation of benign immune cells and stromal cells to produce pro-tumorigenic growth and survival factors. At the same time, the malignant T cells express immunosuppressive factors that foster an immune-privileged tumor site in part by direct inhibition of non-malignant T cells and induction of tolerogenic macrophages and dendritic cells.

Although it is not known exactly how the malignant T cells gain the capacity to impose these changes on the inflammatory environment, a series of recent data suggests that endogenous changes in the malignant T cells, including somatic mutations, somatic copy number variations, and epigenetic deregulation, may play a critical role [19–25, 60, 151, 152]. Exogenous factors such as bacterial colonization and infection may also drive activation of pro-oncogenic mechanisms and inhibit anti-tumor immunity. In particular, *S. aureus* and its toxins appear to possess the ability to stimulate malignant exploitation of non-malignant T cells in a manner which leads to activation of the Jak/Stat pathway and release of immune regulatory cytokines such as IL-10. Dysregulation of the Jak/Stat pathway emerges as a central event in the development of malignant inflammation in CTCL and, indeed, evidence from various other cancers also points towards a central role for certain Stat proteins (e.g., Stat3) in sustaining chronic inflammation while antagonizing anti-tumor responses [153]. Taken together, these findings suggest that disease progression is associated with, and at least partly driven by, changes in the tumor-associated inflammatory environment that are elicited by the malignant T cells. We suspect that once established, malignant inflammation will stimulate tumor growth and foster deepening of the malignant T cell phenotype, thereby further enhancing pro-tumorigenic inflammatory processes that may result in a self-perpetuating vicious circle accelerating cancer progression.

## Malignant inflammation in solid cancers

The importance of the tumor-associated inflammatory environment is now more and more recognized in many solid tumor contexts and models [154–156]. In particular, a bias in Th1/Th2 cell ratio and/or changes in the circulating cytokine profile are increasingly associated with many solid tumor types, including prostate, oral, ovarian, pancreatic, cervical, colorectal, and breast cancers [157–165]. There is also evidence that the tumor-immune environment influences therapeutic responses. Breast cancer is, for example, known to harbor a pro-inflammatory milieu often associated with obesity and increased PGE2 levels [166]. However, patients harboring an increased number of Th1 relative to Th2 cells typically experience superior outcomes [159]. Related to this, a loss of anti-HER2 Th1 cells in breast cancer correlates with poor response to HER2-targetted therapies [161]. The mechanisms underpinning the shift from a Th1 to a Th2 response in non-CTCL cancers are subject of intense research. Recently, it has been shown that polycomb repressive complex mediates epigenetic silencing of the Th1 chemokines, CXCL9 and CXCL10, in ovarian and colorectal cancers, thereby reducing recruitment of CD8^+^ T cells [162, 165]. Ovarian and colorectal cancer patients with increased numbers of intra-tumoral CD8^+^ T cells or Th1 cells consistently experience better outcomes [158, 162, 165]. Therefore, epigenetic-targeting therapies may potentiate the sensitivity to immuno-therapies by modulating the inflammatory environment. As indicated by these examples, malignant inflammation, as defined here, may also be of importance in solid cancers, suggesting that some of the mechanisms described in CTCL may be of general relevance in cancer.

## Conclusions

In conclusion, recent as well as earlier data support our hypothesis that the malignant T cells are key drivers of the inflammatory changes observed during disease progression in CTCL, and that these changes inhibit anti-tumor immunity while promoting tumor growth. If correct, this hypothesis predicts that the most effective treatment of progressive and advanced disease should rationally combine therapeutics that directly target the malignant T cells with drugs that (i) enhance cellular immunity, (ii) neutralize immune evasive mechanisms, (iii) inhibit the pro-tumorigenic environment, and (iv) eliminate pro-oncogenic bacteria such as enterotoxin-producing *S. aureus* in infected patients. Such combined therapeutic strategies would perhaps give a realistic hope for effective treatment, or even a cure, of this devastating disease.
